# Inter-rater Reliability of Physiological and Pressure Pain Threshold Measurements During the Assessment of the Requiescence Technique in Healthy Young Adults

**DOI:** 10.7759/cureus.113004

**Published:** 2026-07-20

**Authors:** Aishwarya Vaidya, Unnati Pandit, Kirti Jadhav

**Affiliations:** 1 Department of Physiotherapy, School of Physiotherapy, D. Y. Patil Deemed to be University, Nerul, IND

**Keywords:** autonomic regulation, intraclass correlation coefficient, pain threshold, relaxation intervention, requiescence technique

## Abstract

Background: Relaxation techniques are widely studied for their influence on autonomic nervous system activity and pain perception. The requiescence technique (RT) is a structured integrative relaxation technique incorporating breathing exercises, guided imagery, colour-based visualisation, gentle movements, and meditation. The present study focused on the reproducibility of physiological and pressure pain threshold measurements obtained during RT assessment rather than on establishing the validity or efficacy of RT itself.

Objectives: This study aimed to assess the inter-rater reliability of heart rate, respiratory rate, and pressure pain threshold measurements at the triceps and trapezius obtained during the evaluation of RT in healthy young adults aged 18-25 years.

Methods: A prospective single-group study with repeated measurements was conducted among 93 healthy participants aged 18-25 years. Heart rate, respiratory rate, and pressure pain threshold at the triceps and trapezius were assessed using standardised procedures. During pressure algometry, pressure was applied perpendicular to the skin at an approximately constant rate of 0.5 kg/cm²/s using a 1 cm² probe tip, and the pressure pain threshold was recorded in kg/cm² at the point when pressure first became painful. A total of 61 participants underwent repeat assessment by a second investigator to evaluate inter-rater reliability. Reliability was assessed using intraclass correlation coefficients (ICC) with 95% confidence intervals.

Results: Heart rate demonstrated poor inter-rater reliability (ICC = 0.492), whereas respiratory rate demonstrated moderate but borderline reliability (ICC = 0.542). Pressure pain threshold measurements demonstrated poor reliability at both the triceps (ICC = 0.226) and trapezius (ICC = 0.492). The triceps estimate was particularly uncertain because its confidence interval included zero. Overall, the findings indicated limited and inconsistent reproducibility, particularly for pressure pain threshold measurements.

Conclusion: The study demonstrated poor inter-rater reliability for heart rate and pressure pain threshold measurements and moderate but borderline reliability for respiratory rate. These findings concern the reproducibility of the measurement procedures and should not be interpreted as evidence of RT validity, efficacy, clinical effectiveness, or intervention reliability. Further studies using larger and more diverse samples, blinded assessors, standardised measurement procedures, repeated assessments, and appropriate pre- and post-intervention analyses are required to confirm the reproducibility of these measurements and to evaluate RT-related physiological and sensory changes.

## Introduction

Pain and stress are complex biopsychosocial events that have a significant impact on human health, functional ability, and well-being. Chronic or recurrent pain is closely linked with emotional suffering, a decrease in quality of life, and impairment of performance in everyday life, and stress is linked to physiological anomie and increased sensitivity to pain. The rising burden of these afflictions has raised the concern of using management strategies beyond pharmacological management. Non-pharmacological interventions have become increasingly popular in recent years because of their safety profile, availability, and possible capacity to treat physiological and psychological contributory factors. Progressive muscle relaxation, mindfulness, and diaphragmatic breathing are examples of relaxation-based techniques that have demonstrated consistent results. These techniques have been linked to positive changes in autonomic control, perceived stress decrease, and better coping capacity among various populations [1-4].

One of the primary mechanisms underlying relaxation-based interventions is their influence on the autonomic nervous system (ANS). The ANS regulates involuntary physiological functions, including vascular tone, heart rate, respiratory rate, and components of pain modulation. Emotional regulation and stress reactivity are also closely associated with autonomic responses. During stress, sympathetic activity commonly increases, and parasympathetic activity decreases, which may contribute to heightened arousal, muscle tension, stress-induced hyperalgesia, and sympathetically maintained pain through altered descending pain-modulatory pathways. This relationship is not strictly linear, since acute sympathetic arousal may transiently increase pain thresholds in some circumstances, whereas sustained or repeated arousal is more commonly associated with increased pain sensitivity. Relaxation methods may attenuate these responses by enhancing parasympathetic activity, reducing excessive sympathetic activation, and supporting autonomic regulation, rather than producing absolute parasympathetic dominance. Due to this responsiveness, the ANS is commonly considered an important physiological marker in interventions designed to reduce stress and modulate pain [5].

Recent research has increasingly examined multimodal relaxation approaches that combine several components rather than relying on a single technique. Such approaches may engage multiple sensory and attentional pathways and support deeper relaxation. The requiescence technique (RT) is a structured integrative relaxation technique incorporating diaphragmatic breathing, guided imagery, colour-based visualisation, gentle movements, and meditation. Conceptually, RT shares features with mindfulness meditation, yoga-based relaxation, and guided imagery. Limited evidence is currently available regarding the reproducibility of physiological and pressure pain threshold measurements obtained during RT assessment [6-8].

Based on findings from a broader body of research on mind-body interventions, integrative interventions are capable of modulating various physiological indicators related to stress and pain management. Improvements in heart rate variability and decreases in blood pressure, as well as changes in the autonomic tone, have been reported as a result of mindfulness-based programs, meditative practices, and relaxation training. The results have been witnessed both in clinical groups and stress-related disorders with chronic pain and in non-clinical groups. These results imply that mind-body therapies can be used in the enhancement of physiological resilience and decrease stress-related autonomic activation. Also, the measures have been linked with alterations in pain perception and pain, proving their possible implementation in nociceptive modulation [9-11].

Pain threshold is more often utilised as an objective measure of nociceptive sensitivity modification. In contrast with the subjective rating of pain, the pain threshold measures can give objective information on the reaction of a person to standardised stimuli. This is usually done using pressure algometry, which is capable of measuring pressure pain threshold at various anatomical sites at acceptable levels. As shown in earlier research, relaxation, meditation, and guided imagery may enhance pressure pain thresholds, which can be interpreted to mean that there is a reduction in pain sensitivity with an increase in the endogenous modulation of pain. It is believed that these changes are acquired as a result of a complex of autonomic regulation, attentional control, emotional modulation, and changes in central pain processing. Accordingly, the measurement of pain threshold offers a significant approach to determining the sensory impact of relaxation-based interventions [12-14]. The reliability of pressure pain threshold measurement depends on standardised probe placement, pressure-application rate, participant response, and assessor technique.

The trapezius and triceps were selected as standardised anatomical sites for pressure pain threshold assessment. The trapezius is commonly associated with stress-related muscle tension, whereas the triceps was included as an accessible upper-limb comparison site that permits consistent algometer positioning and repeated measurement. Heart rate and respiratory rate were assessed alongside pressure pain threshold as indicators of autonomic activity; reductions in these parameters may reflect decreased physiological arousal and increased parasympathetic influence [15-17]. Although evidence regarding mind-body relaxation interventions is increasing, RT has not been adequately examined, particularly in healthy young adults. Further evaluation of its measurement properties is required before comparative or clinical application. The present study focused on the inter-rater reproducibility of these physiological and sensory measurements rather than on establishing the efficacy, validity, or therapeutic reliability of RT.

Objectives of the study

This study aims to assess the inter-rater reliability of heart rate, respiratory rate, and pressure pain threshold measurements at the triceps and trapezius obtained during the evaluation of RT in healthy young adults aged 18-25 years. The study focuses on the reproducibility of these measurement procedures and does not seek to establish the validity, efficacy, or therapeutic reliability of RT itself.

## Materials and methods

Study design

This study was conducted at the School of Physiotherapy, D. Y. Patil Deemed to be University in Nerul, Navi Mumbai, India. The purpose of this prospective single-group study with repeated measurements was to assess the inter-rater reliability of heart rate, respiratory rate, and pressure pain threshold measurements obtained during the evaluation of RT. The study was conducted under controlled conditions to standardise screening, intervention delivery, participant positioning, and outcome assessment. A total of 93 healthy young adults aged 18-25 years were recruited. Inter-rater reliability was evaluated in a subsample of 61 participants who underwent repeat assessment by a second investigator. Although measurements were obtained across the four-week intervention period, the present analysis focused on measurement reproducibility rather than establishing the efficacy or validity of RT as an intervention.

Participants

Healthy individuals aged 18-25 years were recruited primarily from a university student population. Convenience sampling from a single university introduced a risk of sampling bias and limited the representativeness of the study population. The sample should therefore be interpreted as a group of healthy young university students rather than as representative of all healthy young adults. Participants were selected according to predefined inclusion and exclusion criteria to reduce medical and psychological confounding. Written informed consent was obtained from all participants.

Eligibility criteria

Inclusion Criteria

Inclusion criteria were healthy young adults aged 18-25 years who were willing to participate and able to provide written informed consent.

Exclusion Criteria

Participants were excluded if they had a history of chronic pain, cardiovascular or respiratory disease, severe psychological illness, pregnancy, substance abuse, previous extensive experience with relaxation techniques, or any contraindication to relaxation-based interventions. Participants were also excluded if they had taken medications capable of altering pain perception or physiological responses, including analgesics, beta-blockers, anxiolytics, sedatives, antidepressants, or stimulants, within 48 hours before testing. Eligibility was assessed using a structured self-reported medical history questionnaire followed by a verbal screening interview conducted by the investigator.

Sampling method and sample size

Convenience sampling was used to recruit eligible university students. Participants were approached through direct investigator-led invitations within the university and were enrolled voluntarily after eligibility screening and the provision of written informed consent. This recruitment approach may have introduced self-selection and sampling bias. The final sample size was 93 participants. The inter-rater reliability subsample comprised 61 participants who were available during the predefined reliability-assessment period and completed same-session assessments by both investigators. No formal a priori power analysis was performed for either the full sample or the reliability subsample. The sample sizes were determined by the number of eligible participants available during the predefined recruitment period and the practical resources required for repeated assessments. The feasibility-based sampling approach limits the statistical precision and generalizability of the findings and is acknowledged as a methodological limitation.

Ethical considerations

The Institutional Ethics Committee (IEC) for Biomedical and Health Research of D. Y. Patil Deemed to be University School of Medicine, Navi Mumbai, approved the study under reference number DYP/IECBH/2023/337. All participants were informed about the study objectives, procedures, and potential risks before enrollment, and written informed consent was obtained after they had an opportunity to ask questions. Confidentiality and privacy were maintained throughout the study. The collected data included participant demographic and eligibility information, heart rate, respiratory rate, pressure pain threshold measurements at the triceps and trapezius, and repeated measurements used for inter-rater reliability analysis. All data were anonymised and stored in password-protected electronic files accessible only to the research team. The data will be retained for five years following the publication of the study and will then be permanently deleted using secure electronic data-destruction procedures. Any paper-based records will be stored in a locked cabinet for the same period and subsequently destroyed by secure shredding.

Intervention: RT

RT was administered as a standardised, audio-guided integrative relaxation technique intended to reduce physiological arousal and promote a calm mental state. Each session followed the same fixed sequence: approximately five minutes of diaphragmatic breathing, sequential muscle relaxation, guided imagery with colour-based visualisation, gentle movements, focused-attention meditation, and gradual return to the resting state. Participants were instructed to inhale gently through the nose, allow abdominal expansion, and exhale slowly without breath-holding. Sequential relaxation progressed from the lower limbs to the face and shoulders, with instructions to identify and release unnecessary muscular tension.

The guided-imagery component involved the visualisation of a quiet natural environment and calming colours, particularly blue and green. Colour-based relaxation was delivered through verbal suggestion within the prerecorded audio rather than exposure to coloured lamps or other external light sources. Participants were instructed to focus on the imagined colours and associate them with calmness, reduced tension, and comfortable breathing. Gentle movements consisted of slow, pain-free movements of the neck, shoulders, upper limbs, and ankles within each participant's comfortable range. The meditation component involved focused attention on breathing and non-judgmental awareness of bodily sensations.

The same prerecorded audio was used for all participants and sessions to maintain consistency of intervention content, sequence, and verbal instructions. Participants completed each session individually while seated in a comfortable chair with back support, both feet flat on the floor, and the upper limbs resting on the thighs. Sessions were conducted in a quiet, adequately ventilated room with minimal external noise, interruption, and visual distraction. An investigator remained present to provide initial instructions, monitor adherence to the audio-guided sequence, observe participant safety, and record discomfort or interruption, but did not provide additional guidance during the recording.

Each session lasted approximately 20-30 minutes, and participants completed eight sessions over four weeks, with two sessions per week. Attendance and session completion were documented by the supervising investigator. All 93 participants completed the scheduled assessments included in the analysis, and no missing outcome measurements were reported. The same room arrangement, audio recording, participant position, session sequence, and measurement procedures were maintained throughout the intervention period to support treatment fidelity and reproducibility.

Outcome measures

The study evaluated two primary outcome domains: pressure pain threshold and physiological parameters. Pressure pain threshold was assessed at the trapezius and triceps muscles using a handheld pressure algometer fitted with a 1 cm² probe tip. The manufacturer and model of the algometer were not recorded in the available study documentation; therefore, the device is described according to its verified technical characteristics. The same algometer and standardised anatomical sites were used throughout the study. Participants remained in the standardised seated position during measurement, and the investigator positioned the probe perpendicular to the skin. Pressure was increased at an approximately constant rate of 0.5 kg/cm²/s until the participant first reported that the pressure sensation had become painful. The corresponding value was recorded in kg/cm² as the pressure pain threshold.

The assessors followed the same instructions for anatomical-site identification, probe placement, pressure direction, application rate, and participant response endpoint. The available study documentation did not specify whether repeated pressure pain threshold trials were performed or averaged; consequently, no unsupported averaging procedure was introduced into the analysis description. Heart rate and respiratory rate were recorded under the same seated and controlled environmental conditions used for pressure pain threshold assessment. Heart rate, respiratory rate, and pressure pain threshold were collected according to the repeated-measures study protocol. The analysis presented in this manuscript focused on the inter-rater reproducibility of these measurements rather than on acute or intersession changes associated with RT.

Procedures

Pre-intervention Assessment

A preliminary screening was conducted to confirm eligibility according to the predefined inclusion and exclusion criteria. The study procedures were explained to all participants, and written informed consent was obtained before data collection. Baseline assessment included measurement of heart rate, respiratory rate, and pressure pain threshold at the trapezius and triceps using a pressure algometer. These measurements were collected according to the repeated-measures protocol, whereas the analysis presented in this manuscript focused on inter-rater reproducibility rather than on baseline-to-post-session change.

Intervention Sessions

The RT sessions were conducted according to a standardised schedule. Participants remained seated in a comfortable chair with back support, both feet placed flat on the floor, and the upper limbs resting comfortably on the thighs throughout the 20-30-minute intervention. Each session began with approximately five minutes of guided deep breathing and muscle relaxation to prepare the participant for the technique. The main RT was then delivered using a standardised prerecorded audio recording. The session concluded with approximately five minutes of post-relaxation guidance to allow a gradual return to the resting state. The same seated position, room arrangement, and session sequence were maintained for all participants to minimise variation in muscle tension and physiological measurements.

Post-session Assessment

Physiological parameters were measured immediately after each intervention session. Respiratory rate, heart rate, and pressure pain threshold at the trapezius and triceps were recorded according to the standardised repeated-measures protocol. The same investigator supervised the intervention sessions and performed the pre- and post-intervention measurements. Outcome assessors were therefore not blinded to the intervention or measurement time point, introducing a potential risk of investigator and measurement bias. Standardised instructions, participant positioning, measurement sequence, algometer placement, and pressure-application procedures were used to reduce assessor-related variability. The analysis presented in this manuscript focused on inter-rater reproducibility of the measurements rather than on immediate or intersession changes following RT.

Reliability testing

Inter-rater reliability was assessed to determine the reproducibility of physiological and pressure pain threshold measurements between investigators. A total of 61 participants underwent reliability testing. This subsample was selected according to participant availability during the reliability-assessment period. No separate a priori power calculation was performed for the reliability subsample, and this was considered a methodological limitation.

Each participant was assessed independently by two investigators during the same study session. The reliability assessment was conducted at the same measurement occasion for both investigators so that differences in heart rate, respiratory rate, and pressure pain threshold primarily reflected assessor-related variation rather than physiological change across separate sessions. The second assessment was performed immediately after the first, following a brief standardised rest interval. Both investigators followed the same procedures for participant positioning, anatomical-site identification, algometer placement, direction and rate of pressure application, heart rate measurement, and respiratory rate measurement. Before data collection, both investigators reviewed and practiced the standardised measurement protocol to ensure consistent administration. The investigators recorded their findings separately and remained blinded to each other's measurements until data collection was completed.

Statistical analysis

Inter-rater reliability was quantified using average-measures intraclass correlation coefficients (ICC) with 95% confidence intervals. An absolute agreement approach was used because the analysis evaluated whether the two investigators obtained comparable numerical measurements rather than merely preserving the relative ranking of participants. ICC was selected instead of Cohen's kappa because heart rate, respiratory rate, and pressure pain threshold were continuous variables. The specific variance-components formulation of the ICC was not restated beyond the information supported by the available statistical output.

ICC values below 0.50 were interpreted as poor reliability, values from 0.50 to 0.75 as moderate reliability, values from 0.75 to 0.90 as good reliability, and values above 0.90 as excellent reliability. The reliability analysis used measurements obtained during the designated same-session inter-rater assessment. No imputation was performed because all measurements included in the reliability analysis were complete.

## Results

Participant characteristics

There were 93 healthy participants aged 18-25 years. The sample included 59 women (63.4%) and 34 men (36.6%). Individual age values were not available in the final analysis dataset; therefore, the mean age and standard deviation could not be calculated retrospectively, and age is reported as the predefined range of 18-25 years. All participants completed the baseline assessment and intervention-related measurements.

Reliability results

Heart Rate

Heart rate inter-rater reliability demonstrated poor reliability (ICC = 0.492; 95% CI: 0.232-0.675), with the wide confidence interval indicating substantial uncertainty and limited robustness of the estimate, as shown in Table 1.

**Table 1:** Inter-rater reliability of heart rate ICC estimates are presented for single-measures and average-measures agreement with 95% CI. The F-test evaluates the null hypothesis that the ICC equals zero. Superscript a indicates that the estimator is the same whether the interaction effect is present or absent; superscript b denotes type A ICC calculated using an absolute-agreement definition; and superscript c indicates that the estimate was calculated under the assumption that the interaction effect was absent because it could not otherwise be estimated. The superscripts are explanatory statistical-output notes and do not indicate levels of statistical significance. CI: confidence interval; df: degrees of freedom; ICC: intraclass correlation coefficient

Measure	ICC^b^	95% CI		F-test with true value 0		
		Lower bound	Upper bound	Value	df1	df2
Single measures	0.195^a^	0.070	0.342	2.472	60	180
Average measures	0.492^c^	0.232	0.675	2.472	60	180

Respiratory Rate

Respiratory rate inter-rater reliability demonstrated moderate but borderline reliability (ICC = 0.542; 95% CI: 0.361-0.678), as shown in Table 2. This estimate should be interpreted cautiously because it lies only slightly above the threshold for poor reliability.

**Table 2:** Inter-rater reliability of respiratory rate ICC estimates are presented for single-measures and average-measures agreement with 95% CI. CI: confidence interval; ICC: intraclass correlation coefficient

Measure	ICC	95% CI
Single measures	0.228	0.124-0.345
Average measures	0.542	0.361-0.678

Pain Threshold at the Triceps

Triceps pressure pain threshold demonstrated poor inter-rater reliability (ICC = 0.226; 95% CI: −0.016 to 0.452), as shown in Table 3. The confidence interval crossed zero and was relatively wide, indicating substantial uncertainty, weak reproducibility, and considerable measurement variability.

**Table 3:** Inter-rater reliability of triceps pressure pain threshold ICC estimates are presented for single-measures and average-measures agreement with 95% CI. CI: confidence interval; ICC: intraclass correlation coefficient

Measure	ICC	95% CI
Single measures	0.068	−0.004 to 0.171
Average measures	0.226	−0.016 to 0.452

Pain Threshold at the Trapezius

Trapezius pressure pain threshold demonstrated poor inter-rater reliability (ICC = 0.492; 95% CI: 0.232-0.675). The wide confidence interval indicates substantial uncertainty and limited consistency between investigators.

## Discussion

The present study examined the inter-rater reliability of heart rate, respiratory rate, and pressure pain threshold measurements obtained during the evaluation of RT, an integrative, non-pharmacological relaxation intervention incorporating breathing exercises, colour-based visualisation, guided imagery, gentle movements, and meditation. The reported analyses primarily evaluated the reproducibility of the measurement procedures between investigators rather than the reliability or validity of RT as an intervention. Although the study involved repeated assessments during a four-week intervention period, the reported statistical analysis did not provide a direct paired evaluation of pre- and post-intervention changes. The findings therefore cannot establish whether RT produced significant physiological or sensory effects.

Heart rate demonstrated poor inter-rater reliability (ICC = 0.492), whereas respiratory rate demonstrated moderate but borderline inter-rater reliability (ICC = 0.542). Pressure pain threshold measurements demonstrated poor reliability at both the triceps (ICC = 0.226) and trapezius (ICC = 0.492). These findings indicate that measurement reproducibility was limited, particularly for pressure pain threshold outcomes. The wide confidence intervals indicate substantial uncertainty around the ICC estimates and do not support describing the measurements as consistently or robustly reproducible. The triceps pressure pain threshold estimate was particularly imprecise because its 95% confidence interval crossed zero, indicating that the observed agreement was weak and uncertain.

The poor reliability of pressure pain threshold measurements may be related to individual variation in pain perception, anatomical differences, algometer positioning, pressure-application rate and direction, participant response time, and assessor handling. Pressure pain threshold is influenced by both biological and procedural factors, and small differences in probe placement or application technique may produce appreciable variation. Anatomical-site marking, assessor training, participant familiarisation, consistent pressure application, equipment calibration, and averaging of repeated measurements may improve reproducibility in future studies.

The heart rate and respiratory rate findings may also reflect the inherent variability of autonomic measurements. Respiratory rate is particularly susceptible to observer effects because participants may consciously or unconsciously alter their breathing pattern when they know that respiration is being monitored. Mindfulness, meditation, and breathing-based interventions have previously been associated with measurable psychophysiological responses, although the magnitude and consistency of these responses vary across participants and assessment conditions [9]. The present findings do not demonstrate clinically acceptable reproducibility of the autonomic measurements and should therefore be interpreted cautiously.

The distinction between measurement reliability and intervention reliability is important. Inter-rater reliability indicates whether different investigators obtain comparable values when assessing the same outcome. It does not determine whether RT produces consistent physiological or sensory responses across participants, sessions, or time points. Evaluating the reliability of RT itself would require repeated administration of the intervention under comparable conditions, followed by analysis of the stability of the intervention-related changes. The current findings therefore reflect the reproducibility of heart rate, respiratory rate, and pressure pain threshold measurements rather than the consistency of the RT response.

The validity of RT was not established by the reported analyses. Validity would require a clearly defined comparator, reference standard, or prespecified demonstration that the intervention produced the expected change in relevant outcomes. Bland-Altman analysis evaluates agreement between measurements and does not independently establish intervention validity. A correlation between intervention-related changes in pressure pain threshold and autonomic parameters was also not evaluated. Demonstrating convergent change would require the calculation of participant-level pre- to post-intervention differences in heart rate, respiratory rate, and pressure pain threshold, followed by the Pearson or Spearman correlation analysis, depending on the distribution and linearity of the data.

The individual components of RT have plausible physiological and psychological mechanisms. Breathing exercises may influence autonomic balance and vagal modulation [6], guided visualisation may support attentional regulation and cognitive disengagement from stressors, gentle movements may reduce muscular tension, and meditation may contribute to emotional regulation and attentional stability [7]. Relaxation-oriented practices may also influence pain perception through attentional, emotional, and autonomic pathways [13]. These mechanisms are based on prior literature and provide only a theoretical rationale for RT; they were not directly tested in this study and should not be interpreted as explanations for the reported reliability findings.

The colour-based component was incorporated through guided visualisation within the prerecorded relaxation protocol. Its specific contribution could not be separated from the effects of breathing, meditation, imagery, or movement. Claims regarding the independent physiological or analgesic effects of particular colours are therefore not supported by the present study. The multimodal structure of RT may support participant engagement, but the current design cannot determine whether the combined intervention is superior to any individual component or to another relaxation technique.

The study population consisted of 93 healthy young adults aged 18-25 years, including 59 women and 34 men, recruited predominantly from a university setting through convenience sampling. This recruitment method introduces sampling and self-selection bias and limits generalisability to other age groups, community populations, and individuals with chronic pain, psychological distress, cardiovascular disease, respiratory disease, or autonomic dysfunction.

The study has several methodological strengths. It used objective physiological and sensory outcome measures, including heart rate, respiratory rate, and pressure algometry, and employed a structured multi-session relaxation protocol delivered through the same prerecorded audio. Participant positioning, environmental conditions, intervention sequence, and measurement procedures were standardised. The inclusion of independent same-session assessments by two investigators also provided preliminary evidence regarding the inter-rater reproducibility of the measurement procedures. Ethical approval, informed consent, and predefined eligibility criteria further strengthened the study's conduct.

The study has several limitations. The principal limitation is that the reported analyses assessed measurement reproducibility rather than the reliability or validity of RT itself. Additional limitations include poor or borderline ICC values, wide confidence intervals, lack of assessor blinding to the intervention and measurement time point, convenience sampling, restricted age range, lack of an a priori power calculation, absence of a control group, and lack of a direct paired analysis of pre- and post-intervention changes. The relationship between changes in pain threshold and autonomic parameters was also not examined. The individual components of the multimodal intervention were not isolated, preventing conclusions regarding the independent contribution of breathing, colour-based visualisation, guided imagery, movement, or meditation. Effect sizes for pre- and post-intervention changes were not reported because those changes were not analysed as the primary outcome in the present manuscript. These limitations prevent conclusions regarding the efficacy, clinical validity, or therapeutic reproducibility of RT.

Future studies should use larger and more diverse samples, blinded outcome assessors, prespecified paired pre- and post-intervention analyses, appropriate control groups, standardised algometry procedures, calibrated equipment, repeated measurements, and predefined thresholds for acceptable reliability and agreement. Repeated administration of RT across separate sessions should be used to determine whether the intervention produces stable and reproducible physiological or sensory responses. Correlation analyses between changes in autonomic parameters and pressure pain threshold should also be incorporated to evaluate whether these outcomes change together. Until such evidence is available, the present findings should be interpreted as preliminary evidence regarding the inter-rater reliability of the measurement procedures used during RT assessment.

## Conclusions

The present study provides preliminary information on the inter-rater reliability of heart rate, respiratory rate, and pressure pain threshold measurements obtained during RT assessment. Heart rate showed poor reliability (ICC = 0.492), respiratory rate showed moderate but borderline reliability (ICC = 0.542), and pressure pain threshold showed poor reliability at the triceps (ICC = 0.226) and trapezius (ICC = 0.492). These findings indicate limited and inconsistent measurement reproducibility, with the greatest uncertainty observed for pressure pain threshold outcomes. The study did not establish the validity, efficacy, therapeutic superiority, or clinical effectiveness of RT, and no comparison was made with single-component relaxation approaches. The findings should therefore be interpreted as preliminary evidence concerning the reproducibility of the measurement procedures rather than the effectiveness of the intervention. Further controlled studies using larger and more diverse samples, blinded assessors, standardised measurement procedures, repeated assessments, and appropriate pre- and post-intervention analyses are required before RT can be recommended for clinical or wellness applications.

## References

[1] Woolf CJ (2022). Pain modulation in the spinal cord. Front Pain Res (Lausanne).

[2] McEwen BS (1998). Protective and damaging effects of stress mediators. N Engl J Med.

[3] Chrousos GP (2009). Stress and disorders of the stress system. Nat Rev Endocrinol.

[4] Sharma M, Rush SE (2014). Mindfulness-based stress reduction as a stress management intervention for healthy individuals: a systematic review. J Evid Based Complementary Altern Med.

[5] Lehrer PM, Vaschillo E, Vaschillo B (2000). Resonant frequency biofeedback training to increase cardiac variability: rationale and manual for training. Appl Psychophysiol Biofeedback.

[6] Pal GK, Velkumary S, Madanmohan Madanmohan (2004). Effect of short-term practice of breathing exercises on autonomic functions in normal human volunteers. Indian J Med Res.

[7] Tang YY, Hölzel BK, Posner MI (2015). The neuroscience of mindfulness meditation. Nat Rev Neurosci.

[8] Varvogli L, Darviri C (2011). Stress management techniques: evidence-based procedures that reduce stress and promote health. Health Sci J.

[9] Grossman P, Niemann L, Schmidt S, Walach H (2004). Mindfulness-based stress reduction and health benefits: a meta-analysis. J Psychosom Res.

[10] Zeidan F, Martucci KT, Kraft RA, Gordon NS, McHaffie JG, Coghill RC (2011). Brain mechanisms supporting the modulation of pain by mindfulness meditation. J Neurosci.

[11] Bernardi L, Sleight P, Bandinelli G, Cencetti S, Fattorini L, Wdowczyc-Szulc J, Lagi A (2001). Effect of rosary prayer and yoga mantras on autonomic cardiovascular rhythms: comparative study. BMJ.

[12] Chesterton LS, Sim J, Wright CC, Foster NE (2007). Interrater reliability of algometry in measuring pressure pain thresholds in healthy humans, using multiple raters. Clin J Pain.

[13] Villemure C, Bushnell CM (2002). Cognitive modulation of pain: how do attention and emotion influence pain processing?. Pain.

[14] Morone NE, Greco CM (2007). Mind-body interventions for chronic pain in older adults: a structured review. Pain Med.

[15] Arendt-Nielsen L, Graven-Nielsen T (2008). Muscle pain: sensory implications and interaction with motor control. Clin J Pain.

[16] Flehr A, Barton C, Coles J (2019). #MindinBody - feasibility of vigorous exercise (Bikram yoga versus high intensity interval training) to improve persistent pain in women with a history of trauma: a pilot randomized control trial. BMC Complement Altern Med.

[17] Koo TK, Li MY (2016). A guideline of selecting and reporting intraclass correlation coefficients for reliability research. J Chiropr Med.

